# Microencapsulated Microbial Seed Coating Could Improve Soil Environment and Maize Grain Yield in Saline Soil

**DOI:** 10.3390/plants13223139

**Published:** 2024-11-07

**Authors:** Qiuyan Huo, Min Gong, Yawen Jiang, Xi Yang, Meng Kong, Jiuxing He, Qiang Zhang, Jiqing Song, Xinzhu Li, Wei Han, Xurong Mei, Guohua Lv

**Affiliations:** 1 Institute of Environment and Sustainable Development in Agriculture, Chinese Academy of Agricultural Sciences, Beijing 100081, China; huoqiuyan2022@163.com (Q.H.); 15650090557@163.com (M.G.); yangxii2022@163.com (X.Y.); hkhkmeng@163.com (M.K.); hikerjx@163.com (J.H.); 2 College of Resources and Environment, Shanxi Agricultural University, Taiyuan 030801, China; jiangyawen2024@163.com; 3 Kingenta Ecological Engineering Group Co., Ltd., Linyi 276720, China; zhangqiang@kingenta.com (Q.Z.); songjiqing@caas.cn (J.S.); gh7756@163.com (X.L.); 4 Shandong Agri-Tech Extension Center, Jinan 250013, China; 5 State Key Laboratory of Efficient Utilization of Agricultural Water Resources, China Agricultural University/Chinese Academy of Agricultural Sciences, Beijing 100081, China

**Keywords:** maize, saline stress, microencapsulation, hormones, yield, microbial diversity

## Abstract

Soil salinization is one of the major challenges for modern agriculture, posing a great threat to soil health and food security. Field experiments were conducted to evaluate the effect of seed coating on soil environment and maize growth in saline soils. Three treatments were applied to maize seeds: coating with a microencapsulated microbial agent (ME), coating with microbial only (MB), and no coating (CK). High-throughput sequencing of soil bacterial and fungal 16S and ITS rRNA genes was performed using the Illumina HiSeq platform to analyze the effects of these treatments on soil bacterial and fungal diversity and community structure. Additionally, the influence of different treatments on endogenous hormones and yield of maize were investigated. It was found that the coating with a microencapsulated microbial agent led to decreases in pH and electrical conductivity (EC), while increasing the content of soil available phosphorus. This coating improved soil microbial diversity, significantly increasing the relative abundance of the main bacteria genera, *Bacillus* (34.9%), and the main fungal genera, *Mortierella* (190.4%). The treatment also significantly enhanced indole-3-acetic acid (IAA) by 51.2%, contributing to improvements in resistance to salt stress. The germination rate increased by 22.9%, the 100-grain weight increased by 12.7%, and grain yield increased by 14.3%. The use of the microencapsulated microbial agent effectively mitigated the adverse effects of salt stress on maize plants. This approach is beneficial for promoting sustainable agriculture in saline soils.

## 1. Introduction

Soil salinization is one of the major challenges facing contemporary agriculture [1] and seriously affects agricultural productivity [2]. According to climate change projections, the number of salt-affected areas will increase in the future due to temperature and sea level rises [3]. Natural factors such as arid climate, high evapotranspiration, low topographic conditions, and groundwater levels can lead to further deterioration of saline soils [4], but secondary salinization of the land caused by irrational human activities, such as excessive use of chemical fertilizers and pesticides, heavy irrigation, and destruction of surface vegetation by overgrazing, are the main factors that exacerbate soil salinization [5,6]. Currently, approximately 1.1 billion hectares of land are negatively affected by salinization globally, and this area continues to grow at a rate of 10% per year [7], including more than 20% of irrigated cropland [8]. There are about 99.3 million hectares of various types of saline land resources in China, of which the total area of saline soils with prospects for agricultural use is nearly 36.7 million hectares [9]. Under the current situation of arable land protection and food security, it is important to develop saline agriculture to achieve better production through sustainable and integrated use of genetic resources.

Soil salinity negatively affects plant physiological and metabolic processes in the form of osmotic stress (early) and ionic toxicity (late) [10]; in particular, the seed germination and seedling growth stages are the most sensitive stages of the plant life cycle [11]. Nowadays, various measures, including physical, chemical, biological, and engineered hydraulic methods, have been taken to improve saline soils in order to increase crop productivity [12,13,14]. Bioremediation can utilize biological metabolic activities to reduce the concentration of toxic and harmful substances in the environment or render them harmless, so as to restore the polluted environment to its original state [15]. Salt-tolerant plants, which grow naturally in saline soils, constitute only about 1% of the total flora [16] and are of insufficient commercial value (staple food production), whereas many crops have a low tolerance to salt and do not satisfy human food needs. Another strategy for bioremediation to mitigate the adverse effects of salt stress and enhance the viability of non-saline crops is the use of salt-tolerant microorganisms [17]. According to a report [18], the introduction of salt-tolerant microorganisms in salt-affected soils can increase the productivity of many crops and improve the soil. Salt-tolerant bacteria improve plant growth and health through a variety of mechanisms, including the activation of antioxidant defense mechanisms in plants [19], increasing the fixation of atmospheric nitrogen or the solubilization of phosphorus, iron, and potassium [20], the secretion of extracellular polysaccharides (EPSs), the production of ACC deaminase [10], the maintenance of homeostasis [21], osmotic pressure accumulation [22], and induced production of phytohormones [17].

A strain of *Pontibacter actiniarum DSM 19842* was isolated from fresh kelp obtained from the seashore of Yantai City, Shandong Province, China, and it was found that the use of *Pontibacter actiniarum* was able to increase wheat resistance to salt stress and reduce the content of oxidizing substances [23]. However, environmental factors (e.g., temperature, humidity, pH, etc.) are key factors affecting bacterial activity [24]. It has also been found that the metabolic activity and tolerance of microorganisms to harsh environments can be enhanced by encapsulating bacteria in miniature hermetic capsules [25]. Immobilization of cells by specific carriers can effectively reduce microbial loss and increase bioavailability compared to direct inoculation of bacterial preparations [26]. 

Maize belongs to the saccharine group of plants and is classified as moderately sensitive to salinity, especially at the germination and seedling stages, more so than at later stages of development [27]. In our previous study, indoor potting experiments using microencapsulation technology with *Pontibacter actiniarum DSM 19842* on wheat seeds were carried out. It was found that the impacts of salt stress on wheat growth were reduced [28]. In this study, the effects of microbial microencapsulated seed coating on maize growth and salt tolerance were researched in the field. The role of marine bacteria in regulating the abundance, composition, and function of native soil microbial communities was further investigated. This study provides new insights into microbially mediated salt tolerance and the interactions between introduced and native microorganisms in the rhizosphere. It may offer novel approaches for enhancing the viability of non-saline crops in saline environments.

## 2. Results

### 2.1. Effect of Different Seed Treatments on Soil Characters and the Inter-Root Microbial Community

#### 2.1.1. Soil Characteristics

Table 1 shows the changes in the soil chemical characteristics under different treatments. The application of microencapsulated seed coating (ME) resulted in notable changes in soil characteristics. Specifically, pH decreased from 8.3 to 8.0, soil electrical conductivity (EC) decreased from 4.2 to 3.3 dS m^−1^, and soil available phosphorus content increased from 21.7 to 27.3 mg kg^−1^. For the microbial coating (MB), only the EC decreased from 4.2 to 3.7 dS m^−1^.

#### 2.1.2. Soil Microbial α-Diversity

**Figure 1:**
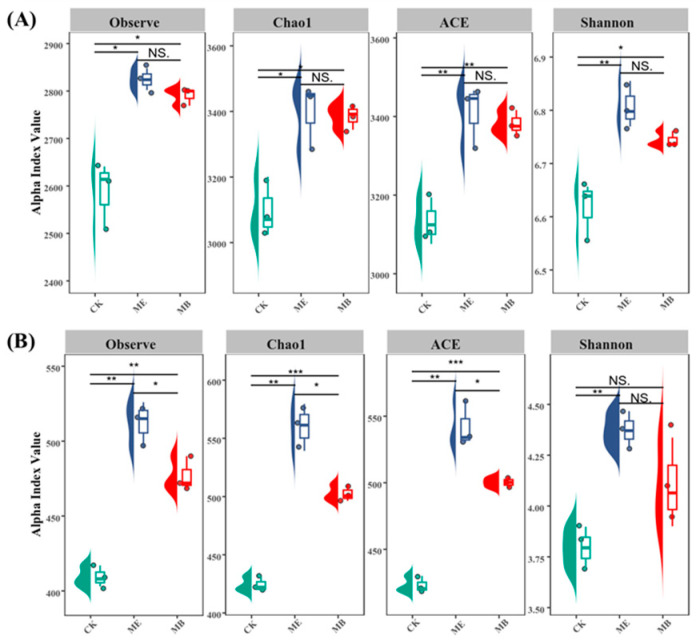
Alpha diversity of soil bacteria (**A**) and fungi (**B**) among different treatment groups. Significant differences in diversity index between different treatments are marked with a star. NS. means *p* > 0.05, * *p* < 0.05, ** *p* < 0.01, *** *p* < 0.001.

Figure 1 shows the results of alpha diversity analysis for both the soil bacterial community and the soil fungal community. For the soil bacterial community (Figure 1A), the observed species and Chao1 indices of the ME were significantly higher than that of CK, with average increases of 9.2% and 9.6%, respectively. The ACE and Shannon indices were obviously higher than those for CK, with increases of 8.9% and 2.8%, respectively. For the MB, the observed species, Chao1 and Shannon index were significantly higher than those for CK, increasing by 7.9%, 9.3% and 1.9%, respectively, and the ACE index was significantly higher by 8.0%. There was no significant difference between the ME and MB. For the soil fungal community (Figure 1B), observed species, Chao1, ACE and Shannon indices for ME were significantly higher than those for CK, with respective increases of 25.2%, 31.9%, 28.1% and 15.4%. For MB, the observed species, Chao1 and ACE indices were significantly higher than those for CK, increasing by 16.5%, 18.5% and 18.2%, respectively. 

#### 2.1.3. Soil Microbial β-Diversity

**Figure 2:**
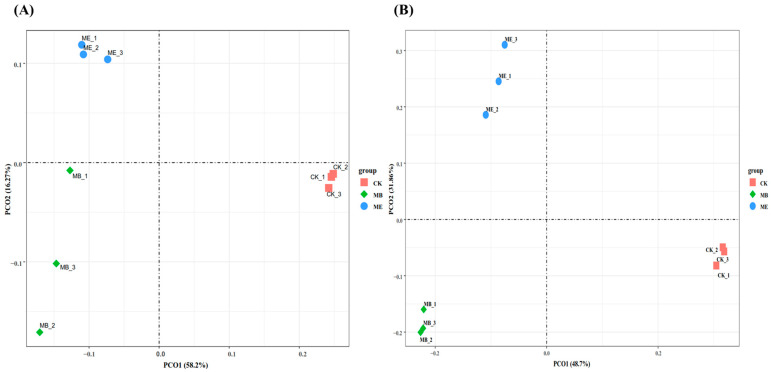
Principal component analysis of bacterial (**A**) and fungal (**B**) communities among different treatment groups.

To further analyze the effects of different treatments on the beta diversity of soil bacterial and fungal communities, principal component analysis (PCA) at the OTU level was performed on nine samples from three treatment groups (ME, MB and CK). As shown in Figure 2, the distribution of samples was more dispersed among the ME, MB and CK groups, indicating that microcapsule and microbial co-treatment, as well as single microbial treatment, caused significant effects on the composition of soil bacterial and fungal communities. Downscaling analysis explained a total of 74.5% of the variation in bacterial community structure, with the first variable axis (PCo1) and the second variable axis (PCo2) explaining 58.2% and 16.3% of the variation in bacterial community structure (Figure 2A). For the fungal community structure, downscaling analysis explained a total of 80.6% of the variation, with PCo1 and PCo2 accounting for 48.7% and 31.9%, respectively (Figure 2B). The results showed that the three treatments were distributed in different quadrants. Samples from the same treatment replicates clustered closer together, which indicated lower variability within each treatment group. The distance between the ME, MB and CK groups was larger, indicating great differences in bacterial flora across different treatments. Additionally, the ME and MB groups had samples distributed farther apart, suggesting greater variability in fungal flora in these two treatment groups.

#### 2.1.4. Soil Microbial Community Composition

The relative abundance of the top 10 phyla was calculated to further interpret the effect of microcapsules on the composition of soil bacterial and fungal communities. At the phylum level, 43 bacterial and 15 fungal phyla were detected in the samples. For soil bacteria, the dominant phylum was Proteobacteria (23.5% ± 3.1%), followed by Acidobacteriota (22.0% ± 2.6%), Chloroflexi (12.4% ± 2.3%), Bacteroidota (7.2% ± 1.8%), and Actinobacteriota (6.8% ± 1.6%). For soil fungi, the major phylum was Ascomycota (46.8% ± 14.9%), followed by Mortierellomycota (14.1% ± 7.5%), Basidiomycota (6.8% ± 4.2%), Chytridiomycota (5.3% ± 3.4%), and Rozellomycota (2.8% ± 2.5%).

**Figure 3:**
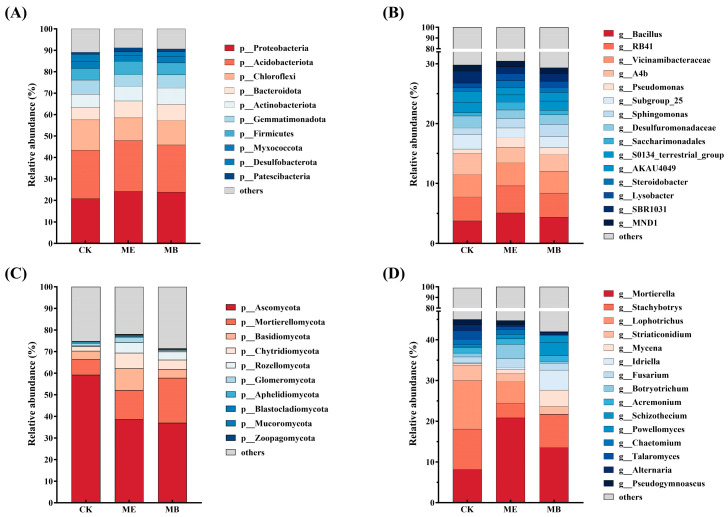
Comparison of relative abundance of the dominant bacterial phyla (**A**) and top 15 classified bacterial genera (**B**) and the dominant fungal phyla (**C**) and top 15 classified fungal genera (**D**) among different treatment groups.

At the bacterial community phylum level (Figure 3A), the relative abundance of Proteobacteria and Patescibacteria in ME was highly significantly higher compared to CK, increasing by 16.1% and 109.8%. Conversely, the relative abundance of Chloroflexi and Desulfobacterota decreased significantly by 33.5% and 94.9%, respectively. In MB, the relative abundance of Chloroflexi and Desulfobacterota decreased significantly by 23.7% and 59.3%, respectively. 

At the fungal community phylum level (Figure 3C), the relative abundance of Mortierellomycota, Rozellomycota, and Chytridiomycota in ME significantly increased by 89.0%, 254.1%, and 126.3%, respectively. In contrast, the relative abundance of Ascomycota decreased significantly by 60.0%. In MB, the relative abundance of Peritrichous Mortierellomycota, Basidiomycota, Rozellomycota and Chytridiomycota increased significantly by 191.0%, 160.2%, 120.4% and 949.3%, while that of Ascomycota decreased significantly by 50.0%.

At the genus level, 670 bacterial and 266 fungal genera were detected. The effects of different treatments on the top 15 classified bacterial and fungal genera in the inter-root soil of maize are shown in Figure 3B,D. The main soil bacterial genera were *Bacillus*, *Vicinamibacteraceae*, *RB41*, *A4b*, and *Pseudomonas*. ME showed a significant increase in *Bacillus, Steroidobacter*, *Saccharimonadales*, and *Pseudomonas*, as compared to CK. The relative abundance of *Bacillus*, *Steroidobacter*, *Saccharimonadales* and *Pseudomonas* was significantly increased by 34.9%, 108.8%, 136.1% and 114.1%, respectively. Conversely, the relative abundance of *Subgroup_25*, *A4b*, *SBR1031* and *Desulfuromonadaceae* was significantly decreased by 52.1%, 34.0%, 73.4% and 40.7%. In MB, the relative abundance of *Sphingomonas* was significantly increased by 91.9%, and *Bacillus* and *Pseudomonas* were highly significantly increased by 15.9% and 60.0%, whereas *Subgroup_25* and *Desulfuromonadaceae* were reduced by 60.0% (*p* < 0.05) and 25.8% (*p* < 0.01). 

The main soil fungal genera were *Mortierella*, *Stachybotrys*, *Lophotrichus*, *Mycena*, and *Idriella*. In ME, the relative abundance of *Mortierella* and *Powellomyce* increased by 190.4% (*p* < 0.01) and 83.5% (*p* < 0.05), whereas significant reductions were observed in *Striaticonidium* (97%, *p* < 0.05), *Stachybotrys* (240.4%, *p* < 0.01), and *Lophotrichus* (195.9%, *p* < 0.01). In MB, the relative abundance of *Mortierella, Mycena, Idriella* and *Schizothecium* increased significantly by 89.4% (*p* < 0.05), 7.9 times (*p* < 0.01), 55.3 times (*p* < 0.01) and 35.9 times (*p* < 0.01), respectively, whereas *Stachybotrys* and *Lophotrichus* decreased significantly by 46.0% (*p* < 0.05) and 217.8 times (*p* < 0.01). 

#### 2.1.5. Soil Microbial Symbiotic Network

**Figure 4:**
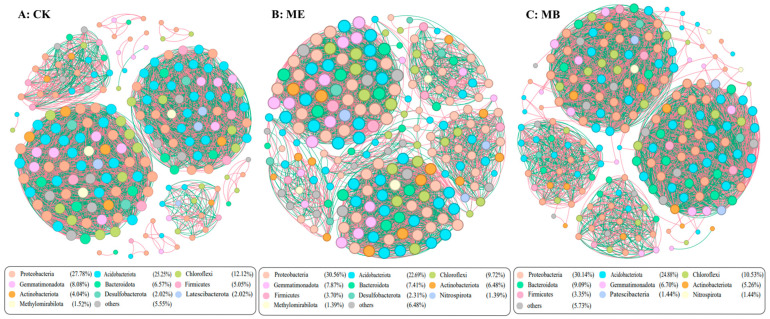
Rhizosphere bacterial network analysis under different treatments.

**Figure 5:**
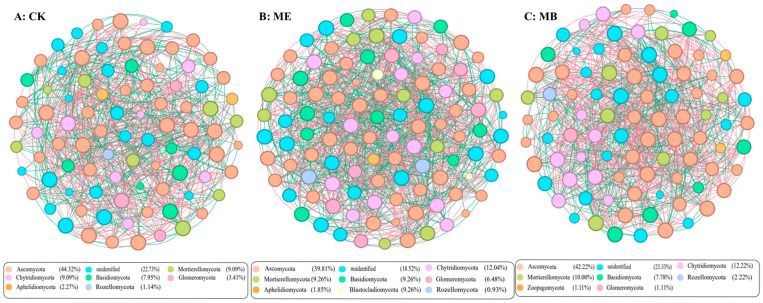
Rhizosphere fungal network analysis under different treatments.

After filtering out low-abundance OTUs (<0.1% abundance), microbial co-occurrence network analysis was constructed using the Spearman correlation matrix (Spearman correlation coefficient r > 0.8, *p* < 0.001). The analysis revealed more positive than negative correlations among OTUs in ME, MB, and CK. In the bacterial community network analysis, ME showed an increase in nodes (5.6%), number of connections (4.1%) and modularity index (11.3%) of the inter-root microbial co-occurrence network (Figure 4B). MB showed an increase in nodes (9.1%) (Figure 4C). In the fungal community network analysis, ME demonstrated a significant increase in nodes (46.1%), number of connections (22.7%) and average degree of nodes (19.1%) of the inter-root microbial co-occurrence network (Figure 5B). MB showed significant increases in nodes (11.2%), number of connections (2.3%) and average degree of nodes (8.7%) (Figure 5C).

### 2.2. Effect of Different Seed Treatments on Maize Growth and Yield

#### 2.2.1. Endogenous Hormones in Maize Seedlings

**Figure 6:**
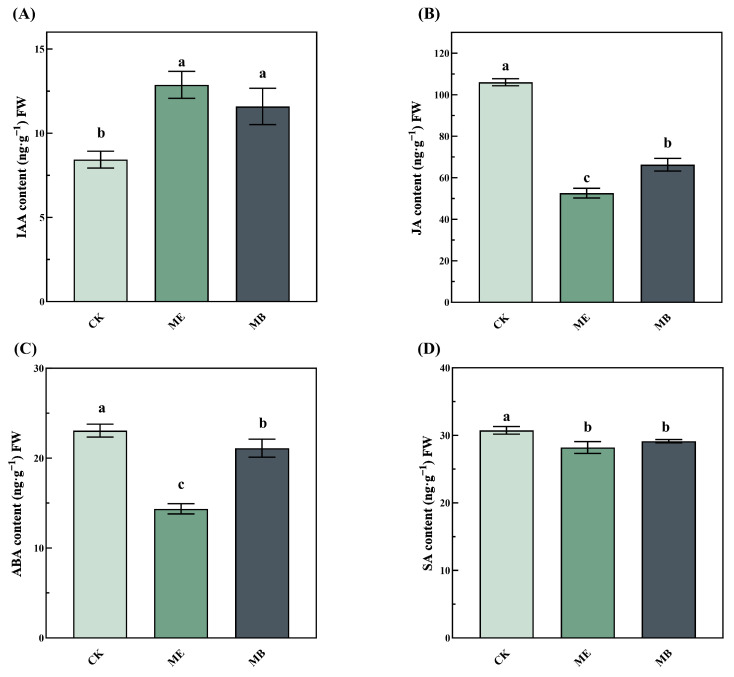
Effects of different seed treatments on endogenous hormones of maize seedlings. (**A**) The indoleacetic acid content; (**B**) the jasmine acid content; (**C**) the abscisic acid content; (**D**) the salicylic acid content. Different letters indicate significant differences by LSD test at *p* < 0.05.

As depicted in Figure 6, IAA content significantly increased by 51.2% in ME and 41.6% in MB (Figure 6A). Conversely, JA content decreased drastically by 101.7% in ME and 59.9% in MB (Figure 6B). The ABA and SA content in seedlings in ME decreased significantly by 60.5% and 8.9%, respectively. Compared with MB, the ABA content in ME decreased significantly by 47.8%, and the JA content also dropped significantly by 26.0%, while there was no significant difference in IAA and SA content.

#### 2.2.2. Maize Growth and Yield

**Figure 7:**
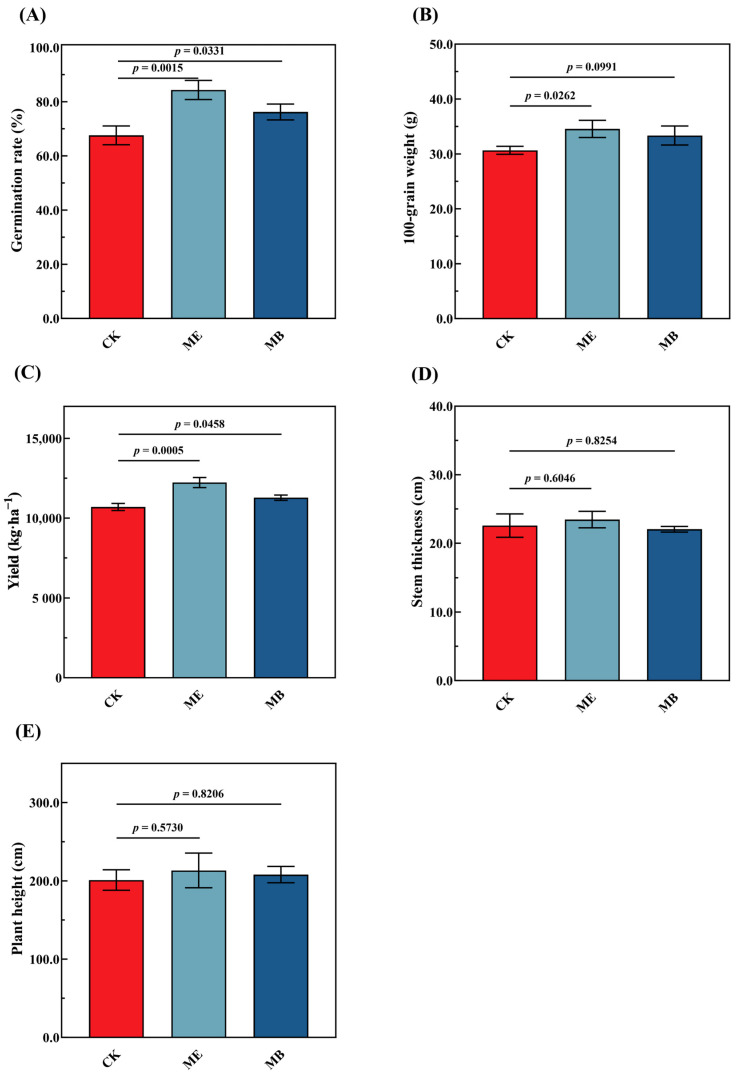
Effects of different seed treatments on maize growth and yield. (**A**) Germination rate; (**B**) 100-grain weight; (**C**) yield; (**D**) plant height; (**E**) stem thickness.

There were great differences in the yield, 100-grain weight and germination of maize. Compared with CK, the germination percentage was high significantly increased by 22.9% in ME and 10.7% in MB (Figure 7A). The 100-grain weight was significantly increased by 12.7% in ME (Figure 7B). Yield was significantly increased by 14.3% in ME and 5.5% in MB (Figure 7C). There was no significant difference in either stem thickness or plant height (Figure 7D,E).

### 2.3. Relationship of Microbial Communities with Maize Growth and Soil Characteristics

**Figure 8:**
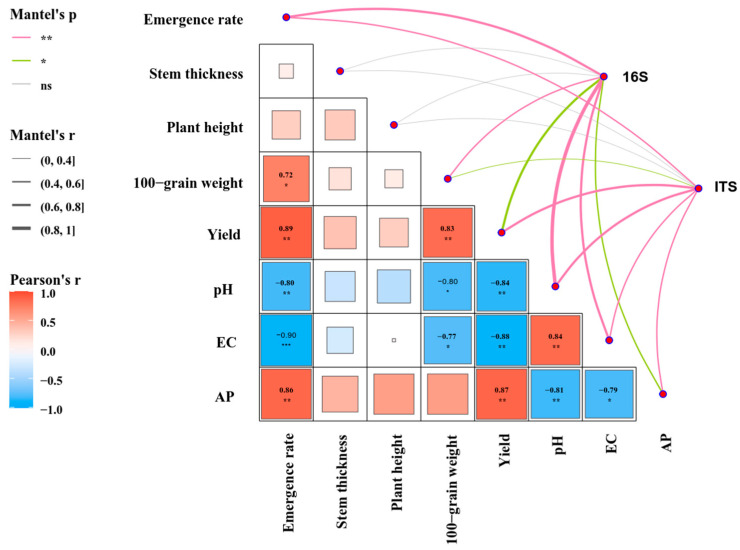
Relationship of microbial communities with yield factors and soil characteristics in maize. ns means *p* > 0.05, * *p* < 0.05, ** *p* < 0.01, *** *p* < 0.001.

Mantel’s test and correlation analysis showed that the germination rate of maize was significantly positively correlated with soil available phosphorus (AP) and yield. It was also significantly positively correlated with 100-grain weight and significantly negatively correlated with soil pH and electrical conductivity (EC). Maize yield was significantly positively correlated with AP and 100-grain weight. Yield was also significantly negatively correlated with soil pH and EC (Figure 8). Six variables were associated with soil bacterial community, with the strongest correlation being with pH, followed by germination rate, EC, AP and yield. Six variables were correlated with soil fungal community, with the strongest correlation being with yield and pH. There was no significant difference between plant height, stem diameter and microbial community of maize.

## 3. Discussion

Salt stress is an abiotic form of stress that negatively affects plant growth and crop productivity [28,29], and phytohormones are essential for plant growth and development. Defense hormones such as ABA, SA, and JA and the growth hormone IAA play important roles in mediating salt stress signals and controlling the balance between plant growth and stress responses [30]. In this experiment, results showed reduced levels of ABA, SA and JA, but IAA levels showed a significant increase in ME. The increase in IAA can promote root growth, expand the surface area and improve root uptake efficiency, which is particularly important in water- or nutrient-constrained environments. This revealed that the application of microencapsulated salt-tolerant bacteria (*Pontibacter actiniarum DSM 19842*) seed coating promoted seed germination through the IAA metabolic pathway. Similar results were also found: inoculation of seeds with *Pseudomonas* spp. enhanced IAA synthesis [31,32]. Usually, the levels of ABA, JA, and SA increase with the intensification of salt stress [33,34]. However, in this experiment, the significant decrease in ABA, JA, and SA could be attributed to the activity of salt-tolerant bacteria present in both ME and MB. Additionally, the most substantial reduction in ABA and JA was observed in ME, indicating that the microcapsules composed of potassium alginate and calcium carbonate effectively helped to alleviate salt stress in maize.

Meanwhile, Zvinavashe [35] et al. found that mixing and applying silk fibroin, alginate and *rhizobacteria* to the seed surface resulted in the formation of more rhizomes in the roots of cauliflower beans, which were able to increase germination and yield. In this study, the capsule wall of microcapsules, calcium alginate, is the main compound of macroalgae, and this compound can promote root growth, enabling plants to absorb more water and nutrients, thus mitigating the effects of salt stress on growth [36,37]. Furthermore, studies have demonstrated [38] that alginate acts as a plant growth bio-stimulant and could attract additional microorganisms in the soil, contributing to the diversity of the soil microbial community.

The alpha diversity indicator is an important indicator for evaluating the diversity of microbial communities and is related to the richness, evenness and diversity of microbial communities [39]. The increase in microbial diversity could improve soil fertility and crop yield [40]. In this experiment, regarding the diversity of bacteria, it was found that there was a significant increase in the observed species, Chao1, ACE, and Shannon indices in ME, indicating that the microencapsulated salt-tolerant bacterial seed coating could increase the abundance and diversity in the inter-root zone. Similarly, regarding the diversity of fungi, ME also showed a significant increase in the observed species, Chao1, and ACE indices, but not the Shannon index, indicating a lesser effect on fungal diversity than on bacterial diversity. This is consistent with the findings of Bruggen [41] that elevated salinity has a greater negative impact on fungi than on bacteria. Therefore, microencapsulated *Pontibacter actiniarum DSM 19842* helped to improve the microbial diversity in saline soil, especially in terms of bacterial diversity.

Microbial community structure is important for soil nutrition. In this study, the phylum Ascomycota was the largest bacterial group, including many salt-resistant bacteria (e.g., *Pseudomonas*, *Sphingomonas*). Recent studies showed that *Pseudomonas* played an important role in promoting plant growth and controlling rhizosphere nutrient accumulation and stress responses [42]. *Sphingomonas* was a common dominant bacterium in saline soil, capable of withstanding barren and harsh environments, exhibiting the characteristics of denitrification and non-symbiotic nitrogen fixation, as well as degrading toxic substances in soil [43]. Additionally, phosphorus-fixing microorganisms were increased, which could secrete organic acids to acidify their surrounding environment and reduce the pH of rhizosphere soil, thus optimizing the microbial environment and alleviating the damage caused by salt stress on plants [44]. The typical genera include *Bacillus*, *Pseudomonas*, *Penicillium*, *Aspergillus*, *Arbusclar mycorrhiza* and *Mortierella*. *Bacillus* had been used a lot to improve the salt tolerance of plants and produce many beneficial substances and functions for plant growth, such as indoleacetic acid, ammonia, nitrogen fixation and phosphorus solution [45]. *Mortierella* could interact with arbuscular mycorrhiza in saline–alkali soils to enhance soil phosphatase activity, thereby promoting plant grow. Furthermore, the ecological niche of pathogenic fungi flora such as Ascomycota was reduced, which contained a variety of plant pathogens that could cause serious damage to crops, such as *Fusarium* spp., *Ustilago* spp., and *Aspergillus* spp. As a result, the decreased soil pH and increased soil available phosphorus content and maize grain yield were observed in this study.

Co-occurrence network analysis revealed microbial interactions, enabling a comprehensive understanding of microbial structure and assembly patterns [46]. The results indicated that the network complexity of bacteria was higher than that of fungi. Fungi were found to be more sensitive to salt stress. The network complexity of fungi inoculated with microencapsulated salt-tolerant bacteria was significantly higher compared to CK, indicating that inoculation with microencapsulated salt-tolerant bacteria had more effects on the fungal microbial community. This suggested that fungi played a more significant role than bacteria in shaping the symbiotic network structure. Microbial communities can adapt to changes in salt stress by enhancing cooperation among microorganisms and altering key microorganisms [47]. In this study, it was observed that ME increased the abundance of *Bacillus*, *Pseudomonas*, *Sphingomonas* and *Mortierella*. Inoculation of *Pontibacter actiniarum DSM 19842* can change the structure of the soil microbial community, increase salt-tolerant bacteria and phosphate-solubilizing bacteria in soil, and further improve the health of the soil environment.

In conclusion, the application of microencapsulated salt-tolerant bacteria (ME) seed coating improved the soil microbial community structure, decreased the pH and electric conductivity, increased the soil available phosphorus and IAA content, and therefore resulted in the highest maize grain yield. This study highlighted the benefits of inoculating microencapsulated salt-tolerant bacteria through seed coating as an efficient bioremediation strategy for saline soils.

## 4. Materials and Methods

### 4.1. Indoor and Field Experiment Design

**Figure 9:**
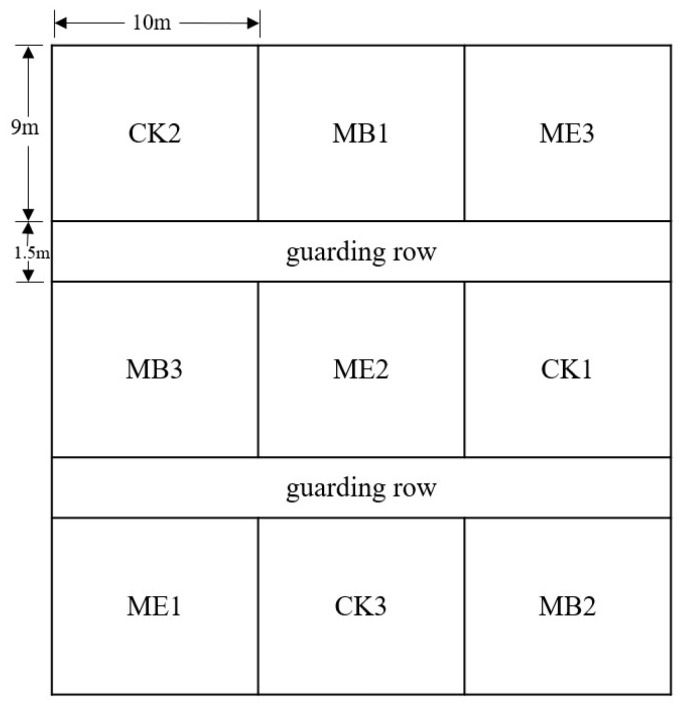
Schematic representation of the layout of maize field trials.

A maize seedling experiment was carried out in a climatic chamber (day/night temperature, 25 °C/20 °C; photoperiod, 12 h; light intensity, 240 μmol m^−2^ s^−1^; humidity, 50%). Field experiments were carried out on coastal saline land, located in Guangrao County, Dongying City, Shandong Province (longitude: E 118°17′, latitude: N 36°56′) (Figure 9). The average annual temperature is 12.9 °C, with annual precipitation ranging from 508.6 mm to 583.0 mm. The soil in the experimental area is loam, and the top soil characteristics in the experimental plots are shown in Table 2. The maize (*Zea mays* L.), variety Zhengdan 958, was sown with three replications of each treatment. The row and plant spacings are 60 and 20 cm, respectively. The same agricultural practices were adopted according to the local experiences. The bacterium used in the indoor Petri dish and field experiments was *Pontibacter actiniarum DSM 19842* [23], which was previously screened and isolated from kelp by our group.

CK involved sterilized and untreated maize seeds. For MB, *Pontibacter actiniarum DSM 19842* was cultured in LB liquid medium for 48 h. The bacterial solution was diluted with sterile water to OD_600nm_ = 0.9, and the sterilized maize seeds were placed inside the diluted bacterial solution for 30 min with stirring under the conditions of 30 °C and 180 rmp [35]. For ME, microcapsules were prepared by endogenous emulsification using *Pontibacter actiniarum DSM 19842* as the capsule core. The above bacterial solution (5 mL) was homogeneously mixed with sterile 2% potassium alginate (50 mL) and calcium carbonate (0.5 g), and the mixture was dispersed into the soybean oil phase containing 1% (*w*/*v*) Span 80 (100 mL), which was emulsified by stirring at 400 rpm for 5 min. Then, stirring was continued in soybean oil (50 mL) containing glacial acetic acid (0.25 mL) for 10 min. After standing and layering, the aqueous phase was removed and centrifuged, washed with phosphate buffer and dried to obtain microcapsules [23].

In the seedling experiment, ten maize seeds from ME, MB and CK were added to Petri dishes with filter paper at the bottom and moistened with NaCl solution at a concentration of 100 mM (11.2 dS m^−1^).

The field experiment was carried out on 30 × 30 m^2^ land, with three treatments set up: maize seeds without coating (CK); maize seeds coated with microencapsulated *Pontibacter actiniarum DSM 19842* (ME); and maize seeds coated with *Pontibacter actiniarum DSM 19842* only (MB).

### 4.2. Pontibacter actiniarum DSM 19842 Features

**Figure 10:**
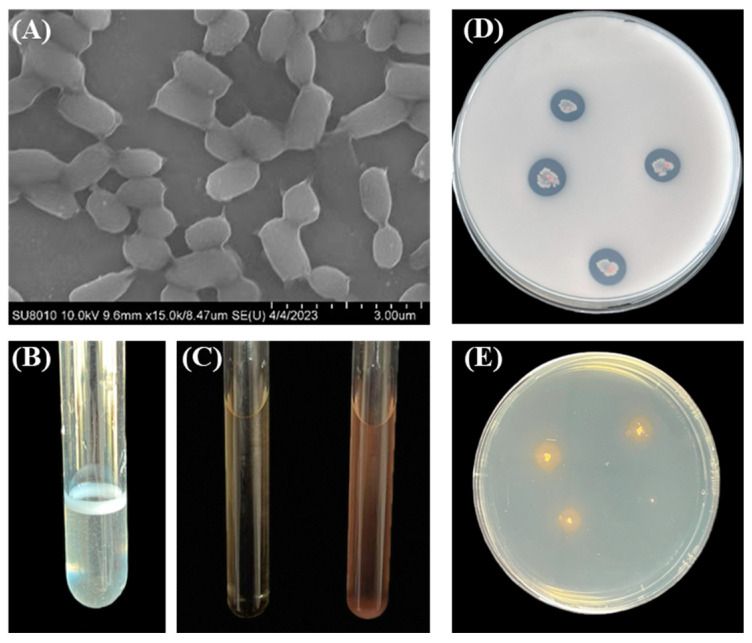
Functional testing of *Pontibacter actiniarum DSM 19842*. (**A**) Scanning electron microscope scans of *Pontibacter actiniarum DSM 19842.* (**B**) Catalase characteristic experiment for catalase positivity. (**C**) IAA grown on tryptophan with nothing added. (**D**): Phosphorus solubilization ability on NBRIP culture medium. (**E**) Siderophore production on CAS culture medium.

*Pontibacter actiniarum DSM 19842* was observed using a JSM-7401F scanning electron microscope (SEM) (JEOL, Tokyo, Japan) (Figure 10A). The surface is smooth; the bacterium is Gram-negative and catalase-test-positive (Figure 10B), and in LB solid medium with added 15% NaCl, it can achieve normal growth. The Salkowski method showed that the supernatant of the strain was pink, indicating that the strain had the ability to produce IAA (Figure 10C). On the NBRIP phosphorous solubilizing medium (Figure 10D), there was a transparent circle around the colony, indicating that the strain had the ability to dissolve inorganic phosphate. On the CAS medium (Figure 10E), the blue color around the colony turned orange-yellow, indicating that the strain was capable of producing iron carriers [48]. In previous studies in our laboratory, it was found that the addition of this strain in a saline–alkali stress environment can promote the growth, development and tolerance of wheat roots [23].

### 4.3. Plant Hormone Detection

After different seed treatments and sowing for 7 days, samples of maize shoots were collected, quickly frozen in liquid nitrogen and ground into powder. Phytohormones (indole-3-acetic acid (IAA), jasmonic acid (JA), abscisic acid (ABA) and salicylic acid (SA)) were detected in the seedlings by UPLC-MS (Ultra-Performance Liquid Chromatography–Mass Spectrometry). The samples were accurately weighed into 50 mg of sample powder and loaded into 2 mL centrifuge tubes; 500 μL of extraction solution (IPA:H_2_O:HCl = 2:1:0.002) was added and the mixture was vortexed shaken for 10 s at 4 °C and 900 rpm for 30 min; then, 1 mL of extraction solution (CHCl_3_) was added and the mixture was vortexed and shaken for 10 s at 4 °C and 900 rpm for 30 min. Then, after centrifugation at 4 °C and 14,000 rpm for 5 min, the two phases were formed. We transferred 1.2 mL of the lower layer of the liquid to be blow-dried under nitrogen at room temperature. Then, 0.1 mL of MeOH was reconstituted, shaken at 4 °C and 900 rpm for 20 min, and centrifuged at 14,000 rpm for 5 min, and the supernatant was extracted, passed through a 0.1 μm filter membrane, loaded into injection vials, and put on the machine for measurement. Ultra-high-pressure liquid chromatography (Waters Acquity UPLC; Allwegene, Chongqing, China) was used. Mobile phase A was water (0.05% formic acid) and mobile phase B was acetonitrile (0.05% formic acid). The column temperature was 35 °C, the sample temperature was 15 °C, and the mobile phase flow rate was 0.3 mL·min^−1^. The sample gradient elution procedure is shown in the table below. Mass Spectrometry was performed with a 5500 Qtrap-MS System (AB SCIEX, Framingham, MA, USA). Lon Source: HESl; spray voltage (-): 3000; capillary temperature: 320; sheath gas: 30; aux gas: 10; spare gas: 5; probe heater temp.: 350; S-Lens RF level: 55. FULL MS-SIM: resolution: 70,000; AGC target: 3 × 10^6^; maximum IT: 100; scan range: 50 to 750m z^−1^ [46].

### 4.4. Determination of Soil Characteristics and Maize Yield Factors

Soil inter-root samples were collected at the most important stage, the silking stage (78 days after sowing), and the topsoil (0–20 cm) samples were randomly selected by using the excavation method from each plot with a consistent density of maize root residue; large particles were removed around the inter-root, and inter-root soils were selected at 2 mm of the inter-root for sampling, with three replications for each treatment, and these samples were stored in self-sealing bags, put on dry ice, transported to the laboratory quickly, and refrigerated at −80 °C for subsequent high-throughput sequencing analyses. At the same time, some soil samples were dried naturally for the determination of soil available nitrogen, phosphorus, potassium, organic matter, pH and electric conductivity. Soil available nitrogen was measured by the alkali diffusion method. Soil available phosphorus was measured by the Olsen method. Soil available potassium was measured by the flame photometer method. Organic matter was determined by the scorching method, electric conductivity (soil/water = 1:5) was determined using a conductivity meter, and soil pH (soil/water = 1:2.5) was measured via the potentiometric immersion method [49]. Field maize emergence was calculated when the third leaf appeared, and stem thickness, plant height, 100-grain weight and yield were measured at harvest.

### 4.5. Soil Microbial DNA Extraction, PCR Amplification and Sequencing

Genomic DNA from soil samples was extracted using the E.Z.N.A.^®^ Soil DNA Kit (Omega Bio-Tek, Norcross, GA, USA). DNA quality and concentration were measured using a Nanodrop2000 (Thermo Fisher Scientific, Inc., Waltham, MA, USA). DNA samples were stored at −20° C for subsequent experiments. The V3-V4 region of the bacterial 16S rRNA gene was amplified with primers 338F (5′-ACTCCTACGGGAGGCAGCA-3′) and 806R (5′-GGACTACHVGGGTWTCTAAT-3′). For fungi, the ITS1 region in the rRNA gene was amplified by PCR using the primers ITS1F (5′-CTTGGTCATTTAGAGGAAGTAA-3′) and ITS2R (5′-TGCGTTCTTCATCGATGC-3′), and the final primers synthesized were amplified on an ABI9700 PCR instrument (Applied Biosystems, Inc., Waltham, MA, USA). Paired-end sequencing was performed based on the Illumina Miseq (San Diego, CA, USA) high-throughput sequencing platform [50,51].

The reaction system of the PCR amplification system included 2 μL of the DNA template, 1 μL of the forward primer (5 μM), 1 μL of the reverse primer (5 μM), 3 μL of BSA (2 ng/μL), 5.5 μL of ddH_2_O and 12.5 μL of 2 × Taq Plus Master Mix (Vazyme Biotech Co., Ltd., Nanjing, China), and the following procedures: pre-denaturation (95 °C, 5 min), denaturation (95 °C, 45 s), annealing (55 °C, 50 s), extension (72 °C, 45 s), final extension (72 °C, 10 min), and holding (4 °C). After the QIIME (v1.8.0) software split the downstream data according to the Barcode sequence, the data were filtered and spliced using the Pear (v0.9.6) software. After splicing, the UPARSE algorithm of the Vsearch (v2.7.1) software was used to cluster the sequences with a similarity threshold of 97% [52]. The sequences were compared using the Silva128 database.

### 4.6. Statistical Analyses

Maize growing paramenters, soil chemical properties and the relative abundance of microorganisms were analyzed by one-way analysis of variance (ANOVA), LSD multiple comparisons (*p* < 0.05), and Pearson correlation analyses using SPSS.25 software and plotted using GraphPad Prism (10.1.2). A-diversity indices were calculated by the MicrobiotaProcess package in R (4.4.1). Differences in bacterial and fungal community structure between samples were compared using principal coordinate analysis with the ‘vegen’ and ‘stats’ packages in R. The Mantel test was carried out using the packages ‘linkET’, ‘patchwork’ and ‘ggplot2’ in R and plotted by the packages ‘vegen’ and ‘stats’. Co-occurrence networks for fungi and bacteria were constructed using the ‘WGCNA’, ‘psych’ and ‘igraph’ packages in R. In order to reduce the bias of correlation coefficients caused by sparse OTU matrices, observed species with a frequency of occurrence lower than 1/6 and an average relative abundance lower than 0.01% were excluded from the network construction, and the correlation level was set at |R| > 0.8, *p* < 0.01. The co-occurrence networks of bacteria and fungi were visualized using Gephi v 10.1 software.

## 5. Conclusions

(1) Improvement of soil environment: Inoculating microencapsulated salt-tolerant bacteria (*Pontibacter actiniarum DSM 19842*) helped to reduce soil pH and electric conductivity and increase soil available phosphorus. The richness and uniformity of bacterial and fungal communities significantly increased in saline soil. This led to an increase in the complexity of microbial co-occurrence networks, revealing its important potential in improving microbial diversity.
(2) Regulation of endogenous hormones: Inoculation with microencapsulated salt-tolerant bacteria significantly reduced the content of ABA, SA and JA in maize seedlings, while increasing the content of the growth hormone IAA, contributing to improvements in resistance to salt stress.
(3) Improvement in maize productivity: Due to improvements in the emergence rate and 100-grain weight, the grain yield of maize was significantly increased, demonstrating that the seed coating played a key role in optimizing the growing environment and enhancing maize productivity.

## Figures and Tables

**Table 1:** The soil chemical characteristics in the 0–20 cm layer.

Treatment	pH	EC dS m^−1^	Organic Matter g kg^−1^	Available Nitrogen mg kg^−1^	Available Phosphorus mg kg^−1^	Available Potassium mg kg^−1^
CK	8.3 b	4.2 b	15.2 a	48.5 a	21.7 b	117.6 a
ME	8.0 a	3.3 a	16.5 a	49.5 a	27.3 a	118.3 a
MB	8.1 b	3.7 a	16.2 a	51.2 a	23.7 b	116.1 a

Notes: Different letters indicate significant differences by LSD test at *p* < 0.05.

**Table 2:** The soil chemical properties in the 0–20 cm layer.

Soil Layers cm	pH	EC dS m^−1^	Organic Matter g kg^−1^	Total Nitrogen g kg^−1^	Total Phosphorus g kg^−1^	Total Potassium g kg^−1^
0–20	8.22	4.51	15.18	1.09	1.03	18.80

## Data Availability

The original contributions presented in this study are included in the article; further inquiries can be directed to the corresponding authors.
